# Lorenz's Operation

**Published:** 1904-04-16

**Authors:** 


### LORENZ'S OPERATION.

We had hoped to be in a position by this time to judge the merits of the so-called bloodless method of repositing the head of the femur in congenital displacement of the hip. Unfortunately this is not the case. The results, as reported by different observers, vary considerably. Thus H. M. Sherman[1] has kept under his observation two children who were operated upon by Lorenz, in San Francisco, in 1902. In both of these failure resulted. When the plaster of Paris was removed the head of the femur was found in each case in the outer part of Scarpa's triangle, and almost immediately subjacent to the skin. In one the condition was so bad that a cutting operation was recommended and carried out. Contrasted with these cases are 33 patients operated upon by Lorenz's method in New York, observed by Dr. V. Gibney and reported by Noble Smith.[2] Dr. Gibney states that of the 33 cases three are cured; the plaster of Paris having been removed, the head of the femur remains where it was placed, and the patients are able to walk without an appreciable limp, the functions of the joint being perfect. Three others have ended in good results; that is, the plaster is removed, the functions of the hip-joint are good, but the limb is not quite so long as the other owing to deformity of the bones forming the joint. Eight cases are in a favourable condition, the bones being in good position, but the patients still wearing plaster of Paris splinting. Another eight are improved. This means that it was impossible to get the head of the bone into an anatomically perfect position, but the position was improved. Four are put down as failures, replacement having been found impossible by reason of the patients' ages. Five are still in the first plaster of Paris dressing, the displacement having been reduced. In one the muscles of the leg became paralysed, but the power is returning, while one patient, a girl aged 12, died. So far as the immediate results go, this series may be taken as showing a high degree of success, but it must always be remembered that the immediate issue of an operation of this kind is not a reliable criterion. Some considerable time must elapse before a particular case can be pronounced cured or even improved, and it may be that many of the cases reported as "cured" will prove ultimately to have derived but little real benefit from the procedure.

[1] Journ. Amer. Med. Assoc., Feb. 27. [2] Clin. Journ., March 9.

